# Novel Analogue of Colchicine Induces Selective Pro-Death Autophagy and Necrosis in Human Cancer Cells

**DOI:** 10.1371/journal.pone.0087064

**Published:** 2014-01-23

**Authors:** Kristen Larocque, Pamela Ovadje, Sinisa Djurdjevic, Mariam Mehdi, James Green, Siyaram Pandey

**Affiliations:** Department of Chemistry & Biochemistry, University of Windsor, Windsor, Onatrio, Canada; Wayne State University School of Medicine, United States of America

## Abstract

Colchicine, a natural product of *Colchicum autumnae* currently used for gout treatment, is a tubulin targeting compound which inhibits microtubule formation by targeting fast dividing cells. This tubulin-targeting property has lead researchers to investigate the potential of colchicine and analogs as possible cancer therapies. One major study conducted on an analogue of allocolchicine, ZD 6126, was halted in phase 2 clinical trials due to severe cardio-toxicity associated with treatment. This study involves the development and testing of novel allocolchicine analogues that hold non-toxic anti-cancer properties. Currently we have synthesized and evaluated the anti-cancer activities of two analogues; N-acetyl-O-methylcolchinol (NSC 51046 or NCME), which is structurally similar to ZD 6126, and (*S*)-3,8,9,10-tetramethoxyallocolchicine (Green 1), which is a novel derivative of allocolchicine that is isomeric in the A ring. NSC 51046 was found to be non-selective as it induced apoptosis in both BxPC-3 and PANC-1 pancreatic cancer cells and in normal human fibroblasts. Interestingly, we found that Green 1 was able to modestly induce pro-death autophagy in these pancreatic cancer cells and E6-1 leukemia cells but not in normal human fibroblasts. Unlike colchicine and NSC 51046, Green 1 does not appear to affect tubulin polymerization indicating that it has a different molecular target. Green 1 also caused increased reactive oxygen species (ROS) production in mitochondria isolated from pancreatic cancer cells. Furthermore, *in vivo* studies revealed that Green 1 was well tolerated in mice. Our findings suggest that a small change in the structure of colchicine has apparently changed the mechanism of action and lead to improved selectivity. This may lead to better selective treatments in cancer therapy.

## Introduction

In Canada, it is estimated that 187, 600 people will be diagnosed with cancer and 75, 500 people will die from cancer in 2013 [1]. Of the vast number of different types of cancer, pancreatic cancer is one of the most deadly, as it is very aggressive, resistant to treatment, progresses rapidly and has a lack of clear symptoms; therefore, it is associated with poor prognosis and late stage diagnosis [2], [3]. Current treatments available for pancreatic cancer include surgery, radiation, and several chemotherapies, including 5-fluorouracil and gemcitabine [4]. Unfortunately, the effectiveness of these treatment options is not long lasting and they are associated with severe adverse side effects, due to non-selective targeting of non-cancerous cells [5]. While much progress has been made in many cancer types, pancreatic cancer cases and deaths are still on the rise [6]. It is of great importance that a selective, safer and non-toxic alternative to current treatment options is developed for those who suffer with pancreatic cancer. Leukemia is another deadly type of cancer that occurs when blood stem cells in the bone marrow develop into abnormal cells. It is estimated to be diagnosed in 5,800 Canadians in 2013, killing 2,600. Although treatment options are available, there is still a need to develop a more effective and safer alternative.

Of particular importance to the survival of cancer cells is their ability to evade programmed cell death (PCD). Specifically, cancer cells are able to bypass apoptosis, PCD type I, involved in homeostatic regulation and development. Apoptosis acts to prevent damaged and mutated cells from proliferating and accumulating [7]. Autophagy, PCD type II, which is a catabolic process that involves the breakdown of damaged cellular components and can also provide energy during times of stress, has also been implicated in the survival of cancer cells. This process of breaking down damaged cellular components provides the cells with materials that can be used to generate energy until the stressor is removed [8], [9]. Due to this process, autophagy has been considered to be only a pro-survival mechanism; recently, however, researchers have found that sustained exposure to stressors can lead to prolonged autophagy and subsequently, cell death [10], [11]. Because of this dual purpose, there is a question as to whether it is more beneficial to inhibit or induce autophagy in order to cause cancer cell death, and researchers are currently exploring both avenues. Lastly, necrosis is a form of pathological cell death that is caused by exposure to infection, toxins, or trauma [12], [13]. Recently, it has been found that necrosis, like apoptosis, can also be programmed and its induction can be another strategy in cancer therapy [14], [15]. With research into cell death induction, researchers are increasingly focused on finding compounds and products, with cancer specific targets, that can lead to the induction of cell death programs in cancer cells selectively, with no induction of cell death in non-cancerous cells, thereby bypassing some of the toxicities associated with current cancer therapies.

**Figure 1:**
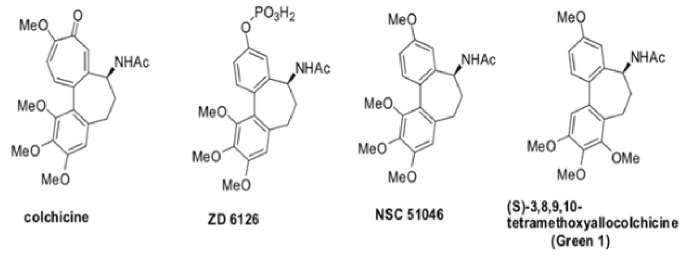
Colchicine and Selected Allocolchicines.

Colchicine is a natural compound that can be isolated from either *Colchicum autumnale* (meadow saffron) or *Gloriosa superba* (glory lily), both of which belong to the lily family [16]. Colchicine is not unfamiliar to the medical world, as it has been utilized in the treatment of gout and has been investigated in many other conditions, including familial Mediterranean fever [17], cirrhosis [18] and Sweet’s syndrome [19]. More recently, allocolchicines (derivatives of colchicine) and other analogues have shown some exciting effects in cancer cells. This is largely due to allocolchicine’s ability to halt mitosis by inhibiting tubulin polymerization into microtubules [16], hindering the progress of cells through the cell cycle and leading to the induction of apoptosis. This inhibition of microtubule formation is especially useful in cancer therapy because cancer cells proliferate rapidly and uncontrollably. One allocolchicine derivative, ZD 6126 which is a pro-drug of N-acetylcolchinol, had some exciting results, as it was able to disrupt the cytoskeleton of tumor endothelial cells and cause apoptosis (Fig. 1) [20], [21]. ZD 6126 caused tumor cell necrosis in *in vivo* mouse models of human lung (Calu-6), colorectal (LoVo and HT-29), prostate (PC-3), ovarian (SKOV-3), and breast (MDA-MB-231) tumors [22]. Unfortunately, this compound was halted in phase 2 clinical trials as a result of associated cardio-toxicity in humans [23]. It is not surprising that this derivative and other derivatives were toxic because tubulin and microtubules are essential to the cell cycle not only in cancer cells, but also in non-cancerous cells; therefore, this non-selective target is one of the reasons that non-cancerous cells are susceptible to apoptosis induced by tubulin targeting agents, leading to the observed side effects [24].

Here we report the anticancer activity of synthetic derivatives of allocolchicine; N-acetyl-O-methylcolchinol (NSC 51046 or NCME), which is structurally similar to ZD 6126, and (*S*)-3,8,9,10-tetramethoxyallocolchicine (Green 1), which is a novel derivative of allocolchicine that is isomeric in the A ring (Fig. 1). In this study, we report the differential mechanisms of two allocolchicine derivatives against leukemia and pancreatic cancer cells caused by slight modifications of their chemical structure. NSC 51046 clearly induced non-selective apoptosis in pancreatic cancer cells (PANC-1 and BxPC-3) and non-cancerous fetal fibroblasts (NFF), while Green 1 caused pro-death autophagy and necrosis selectively in pancreatic cancer cells (PANC-1) and acute T cell leukemia (E6-1 or Jurkat), while having little effect on non-cancerous human fibroblasts (NHF). Furthermore, unlike colchicine, ZD 6126 and NSC 51046, Green 1 does not appear to target tubulin and has a different molecular target. It is very interesting that a small change in the structure of allocolchicine has changed the mechanism of action and lead to improved cancer selectivity. These findings may lead to the development of better, more selective chemotherapeutics for cancer treatment.

## Materials and Methods

### Cell Culture

The human cancer cell lines that were used in this study were pancreatic epitheloid carcinoma (PANC-1; Cat. No. CRL-1469), pancreatic adenocarcinoma (BxPC-3; Cat. No. CRL-1687) and acute T cell leukemia, clone E6-1 (Jurkat; TIB-152), which were all purchased from the American Type Culture Collection (ATCC, Manassas, VA). The normal cell lines used in this study were normal human fibroblasts (NHF; Cat. No. AG09309) and normal fetal fibroblasts (NFF; Cat. No. AG0443), which were both purchased from the Coriell Institute for Medical Research, Camden, NJ. PANC-1 cells were grown in Dulbecco’s Modified Eagle’s Medium (Thermo Scientific, Waltham, MA, USA), supplemented with 10% fetal bovine serum (FBS), (Gibco BRL, Mississauga, ON, Canada) and 10 mg/mL gentamicin (Gibco BRL, Mississauga, ON, Canada). BxPC-3 and Jurkat cells were grown in RPMI-1640 medium (Sigma-Aldrich, ON, Canada), supplemented with 10% FBS (Gibco BRL, Mississauga, ON, Canada) and 10 mg/mL gentamicin (Gibco BRL, Mississauga, ON, Canada). NHF and NFF cells were grown in Minimal Essential Medium with Earle’s Balanced Salts (MEM/EBSS), (Thermo Scientific, Waltham, MA, USA) supplemented with 15% FBS Non-Essential Amino Acids (Gibco BRL, Mississauga, ON, Canada), and 10 mg/mL gentamicin (Gibco BRL, Mississauga, ON, Canada). All cell lines were grown and maintained in a Forma Scientific CO_2_ incubator equipped with a HEPA filter (Forma Scientific Inc, Marietta, Ohio) at 37°C, 95% humidity and 5% CO_2_.

### Cell Treatment

Allocolchicine derivatives, N-acetyl-O-methylcolchinol (NSC 51046) and (*S*)-3,8,9,10-tetramethoxyallocolchicine (Green 1) were used as the key compounds for this study [25]. Cells were treated with increasing concentrations and durations of NSC 51046 and Green 1, reconstituted in dimethylsulfoxide (Me_2_SO). Prior to treatment, stock concentrated compounds were further diluted in phosphate buffered saline (PBS). In addition to allocolchicine derivatives, colchicine (Sigma-Aldrich, ON, Canada) was used to compare effects of allocolchicine derivatives to the already known mechanistic efficacy of colchicines. Paclitaxel (Sigma-Aldrich, ON, Canada) and Paraquat (PQ), (Sigma-Aldrich, ON, Canada) were used as positive controls in various experiments.

### Assessment of Efficacy of Green 1 and NSC 51046

#### WST-1 assay

To measure cell viability, WST-1 dye ([2-(4-iodophenol)-3-(4-nitrophenyl)-5-(2,4-disulfophenyl)-2*H*-tetrazolium, Roche Diagnostics, Mannheim, Germany) was used. This dye is reduced to formazan in the presence of metabolic enzymes, which are indicative of active metabolic activity in viable cells. The amount of formazan can be measured through absorbance at 450 nm. Equal number of cells were seeded in 96-well plates (PANC-1∶4,000 cells/well; NHFs: 4,000 cells/well), with a total volume of 100 µL. Following attachment, the cells were treated with increasing concentrations of Green 1 or NSC 51046. At the desired time point, the WST-1 dye was added to each well and incubated for 4 hours at 37°C. Using a Wallac Victor^3^™ 1420 Multilabel Counter (PerkinElmer, Woodbridge, ON, Canada), absorbance values were measured at 450 nm. The absorbance values of the treated cells were expressed as a percentage of the absorbance values of the control.

#### Trypan blue exclusion assay

To quantify the percentage of dead cells, trypan blue cell impermeable dye was incubated with treated cells [26]. A 1∶1 mixture of cell suspension and trypan blue dye (Sigma-Aldrich, ON, Canada) was pipetted into a hemacytometer (Hausser Scientific, Horsham, PA) and the number of cells (dead cells stained blue and viable cells remained unstained) were manually quantified. The number of dead cells was expressed as a percentage of the total number of cells. Results were analyzed using GraphPad Prism 6.0 and reported as the mean ± SD of two independent experiments.

### Hoechst Staining

In order to visualize nuclear morphology and the induction of apoptosis, Hoechst 33342 dye (Molecular Probes, Eugene, OR, USA) was used to stain the nuclei. Following treatment with either Green 1 or NSC 51046, cells were incubated with 10 µM of the Hoechst 33342 dye for 10 minutes at 37°C. Images were obtained using a Leica DM IRB inverted fluorescence microscope (Wetzlar, Germany) at 400X magnification.

### Annexin V Binding Assay

In order to determine if NSC 51046 and colchicine were inducing apoptosis, an Annexin V binding assay was used. Following treatment, PANC-1 cells were collected, washed in PBS and resuspended in 50 µL Annexin V binding buffer (10 mM HEPES, 140 mM NaCl, 2.5 mM CaCl_2_, ph 7.4). Cells were then incubated with Annexin V AlexaFluor-488 Conjugate (1∶20) (Invitrogen, Canada), 10 µM Hoechst 33342 dye (Molecular Probes, Eugene, OR, USA) and 1 µg/mL propidium iodide (Sigma-Aldrich, ON, Canada) for 15 minutes at 37°C. Images were taken at 400X magnification using the Leica DMI6000 fluorescent microscope (Leica Microsystems, Wetzlar, Germany).

### MDC Assay

To detect autophagy, monodansylcadaverine (MDC; Sigma-Aldrich, ON, Canada) was used. MDC is incorporated into autophagic vacuoles, producing a punctate stain that is observable through fluorescence microscopy. Cells were grown on coverslips and, following overnight incubation, were treated with varying doses of Green 1. Subsequent to treatment, cells were incubated with a final concentration of 0.1 mM MDC (diluted in PBS) for 35 minutes at 37°C. For further confirmation of loss of cell membrane integrity and the induction of cell death, cells were counter-stained with the cell membrane impermeable nuclear stain, propidium iodide at 1 µg/mL (Sigma-Aldrich, ON, Canada) to confirm the induction of cell death. The images were obtained using a Leica DM IRB inverted fluorescence microscope (Wetzlar, Germany) at 400X magnification.

### Cellular Lysate Preparation

Following treatment with 5 µM Green 1 for various time periods, cells were collected, washed in PBS and incubated in 0.1% NP40 buffer (20 mM Tris HCl, 100 mM NaCl, 5 mM EDTA) for 25 minutes on ice (vortexing every 5 minutes, for 15 seconds). The sample is then centrifuged at 3000 rpm for 5 minutes at 4°C. The supernatant (containing the lysed cellular material) is stored at −20°C until use.

### Western Blot

The protein samples were separated using SDS-PAGE. Electrophoresed proteins were then transferred to a nitrocellulose membrane and blocked with 5% w/v milk TBST (Tris-Buffered Saline with Tween-20) solution. The membranes were then probed with primary antibodies in 2% w/v milk TBST overnight at 4°C for microtubule-associated protein1 light chain 3 (LC3) raised in rabbit (1∶500) (Novus Biologicals, Cat. No. NB100–2220, Littleton, CO, USA); β-Actin raised in mouse (1∶1000) (Santa Cruz Biotechnology, Inc., Cat. No. sc-81178, CA, USA); Beclin-1 raised in rabbit (1∶1000) (Santa Cruz Biotechnology, Inc., Cat. No. sc-11427, CA, USA). Following incubation, membranes were washed in TBST and incubated an anti-mouse (1∶2000) or an anti-rabbit (1∶2000) horseradish peroxidase-conjugated secondary antibody (Abcam, Cat. No. ab6728 & ab6802, Cambridge, MA, USA) in 2% w/v milk TBST for 1 hour at room temperature. Following washes in TBST, bands were visualized with enhanced chemiluminescence reagent (Sigma-Aldrich, ON, Canada) and densitometry was performed using ImageJ software.

### Tubulin Polymerization Assay

To observe the effect of allocolchicine derivatives on tubulin polymerization, a tubulin polymerization assay kit (Cytoskeleton Inc. Cat. No. BK006P) was used. A 96-well plate was pre-warmed at 37°C for 30 minutes prior to starting the assay. Control wells received general tubulin buffer (80 mM PIPES pH 6.9, 2 mM MgCl_2_, 0.5 mM EGTA), and the other wells received general tubulin buffer and either Green 1, NSC 51046 or colchicine (Sigma-Aldrich, ON, Canada). All the wells (including control) were then incubated with 3 mg/mL tubulin in tubulin polymerization buffer (80 mM PIPES pH 6.9, 2 mM MgCl_2_, 0.5 mM EGTA, 1 mM GTP and 10.2% glycerol). The OD_340_ was measured every minute for one hour. Polymerization curves were generated using GraphPad Prism 6.0. Results are representative of three independent experiments.

### Mitochondrial Isolation and Measurement of Reactive Oxygen Species (ROS) Production

Mitochondria were isolated from untreated PANC-1 cells to investigate the effect of Green 1 on ROS production and the possibility of the mitochondria as a target of Green 1. Cells were first washed twice in cold PBS, resuspended in hypotonic buffer (1 mM EDTA, 5 mM Tris-HCl, 210 mM mannitol, 70 mM sucrose, 10 µM Leu-pep, 10 µM Pep-A, 100 µM PMSF), homogenized, then centrifuged at 3000 rpm for 5 minutes at 4°C to pellet the nuclear fraction. The supernatant was centrifuged at 12,000 rpm for 15 minutes at 4°C. The cytosolic supernatant was disposed of and the pellet (containing mitochondria) was resuspended in cold reaction buffer and treated with 250 µM paraquat (PQ) as a positive control, 2.5 µM Green 1 or 10 µM colchicine. Amplex Red (Molecular Probes, Eugene, OR), along with horse-radish peroxidase was used to measure mitochondrial ROS production. The mitochondria that were isolated as aforementioned were resuspended in cold hypotonic buffer and 20 µg of protein was added to each well in a black 96-well opaque plate. Fluorescence was measured (Ex. 560 nm and Em. 590 nm) every 5 minutes for 5 hours using a spectrofluorometer (SpectraMax Gemini XS, Molecular Devices, Sunnyvale, CA). Readings were analyzed using GraphPad Prism 6.0 and expressed as relative fluorescence units (RFU) per microgram of protein and represent data obtained from three independent experiments.

### *In Vivo* Assessment of (*S*)-3,8,9,10-tetramethoxyallocolchicine (Green 1)

All procedures involving animals were carried out in accordance with the Canadian Council for Animal Care guidelines and approved by the University of Windsor’s Animal Care Committee. Six week old male CD-1 nu/nu mice were obtained from Charles River Laboratories and housed in constant laboratory conditions of a 12-hour light/dark cycle, in accordance with the animal protocols outlined in the University of Windsor Research Ethics Board- AUPP#: 10–17. Following acclimatization, the mice were separated into two main groups, one group was injected subcutaneously in the right and left hind flanks with Me_2_SO in PBS (10 µL in 200 µL PBS), while the second group received subcutaneous injections of Green 1 (10 mg/kg/day for a total volume of 10 µL Green 1/200 µL PBS) three times a week for a period of one month. To assess toxicity while the mice were alive, body weights were measured three times a week for the duration of the study. Following the period of study, the animals were sacrificed and their organs and tissues (liver, kidneys and heart) were obtained and stored in 10% formaldehyde for immunohistochemical and toxicological analysis.

### Hematoxylin & Eosin (H & E) Staining

Mice organs were fixed in 10% formaldehyde, following which they were cryosectioned into 10 µm sections and placed on a superfrost/Plus microscope slides (Fisherbrand, Fisher Scientific). Sections of organs were stained according to a standardized H & E protocol [27].

### Statistical Analysis

Results are expressed as a mean (SD). Statistical analysis was performed using a 2-way ANOVA test with GraphPad Prism 6.0 software.

## Results

### Effect of Allocolchicine Derivatives on the Viability of Cancer Cells

**Figure 2:**
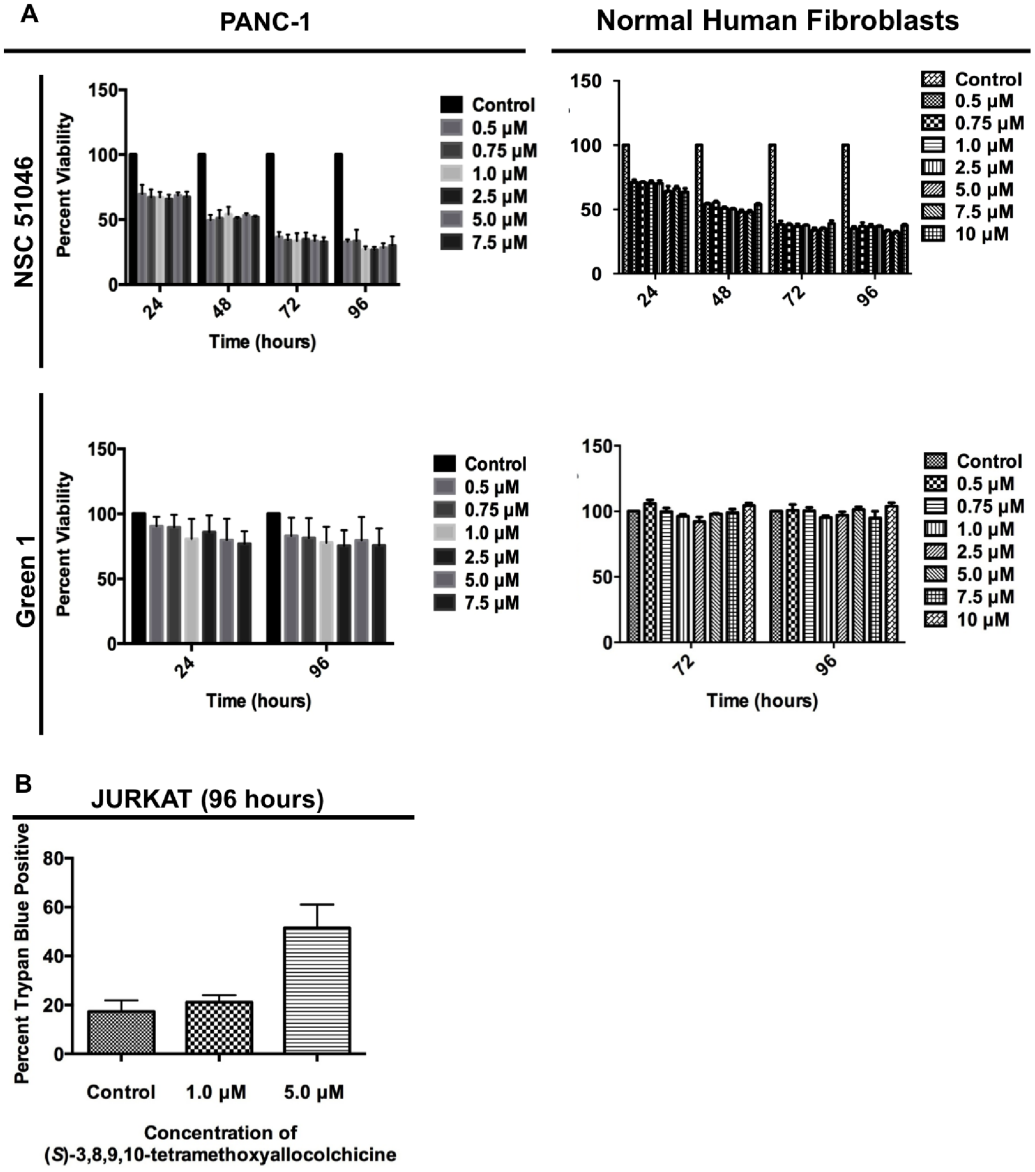
Green 1 Selectively Reduces the Viability of Cancer cells. (A) Pancreatic cancer cells (PANC-1) and normal human fibroblasts (NHFs) were treated with increasing concentrations of Green 1 and NSC 51046 and following treatment at the indicated time points, cells were incubated with WST-1 cell viability dye for 4 hours. Absorbance was read at 450 nm and results were expressed as a percent of the control. Values are expressed as mean ± SD from quadruplicates; (i) PANC-1 treated with NSC 51046 (Interaction between each concentration: ****p<0.0001; Control versus column factor: ****p<0.0001); (ii) NHF treated with NSC 51046 (Interaction between each concentration: ****p<0.0001; Control versus column factor: ****p<0.0001); (iii) PANC-1 treated with Green 1 (Interaction between each concentration: p = 0.9955; Control versus column factor: ****p<0.0001); (iv) NHF treated with Green 1 (Interaction between each concentration: p = 0.3642; Control versus column factor: *p = 0.0370) (B) Following treatment of human acute T cell leukemia cells (Jurkat) with Green 1, a trypan blue excluding assay was carried out. Cells were stained with trypan blue cell membrane impermeable dye and the numbers of trypan blue positive cells were obtained using a hemocytometer. Results are expressed as percent of trypan blue positive cells, indicating dead cells with non-intact cell membranes. Values are expressed as mean ± SD from two independent experiments.

For the purpose of this study, the viability of pancreatic cancer cells (PANC-1) was measured, following treatment with Green 1 or NSC 51046, using the WST-1 cell viability assay. Following a 4-hour incubation with the WST-1 dye, absorbance was read at 450 nm. In parallel, non-cancerous normal human fibroblasts (NHFs) were used as the normal cell counterpart in this study to ascertain the selectivity of the compounds to cancer cells. Our results show that both NSC 51046 and Green 1 were able to reduce the viability of PANC-1 cells. However, it was observed that Green 1 was selective to cancer cells, as the viability of PANC-1 cells was modestly reduced while the viability of NHFs remained unaffected. On the other hand, treatment with NSC 51046 led to the reduction of viability in both cancer and non-cancerous cells (Fig. 2A). Furthermore, Green 1 treatment decreased the proliferation of human T cell leukemia cells and decreased cell membrane integrity, as evidenced by the increase in trypan blue positive cells observed following treatment (Fig. 2B). Collectively, these results indicate that Green 1 is selective towards cancer cells.

### Distinct Modes of Cell Death Induction by Allocolchicine Derivatives

**Figure 3:**
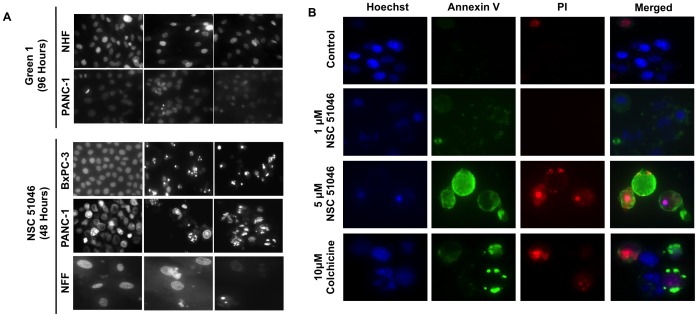
NSC 51046 Induces Non-Selective Apoptosis. Following treatment with either Green 1(A) Pancreatic cancer cells (BxPC-3 and PANC-1) and normal human and fetal fibroblasts (NHFs and NFFs) were incubated with Hoechst 33342 dye to characterize nuclear morphology and detect the induction of apoptosis. Brightly stained, condensed nuclei accompanied by apoptotic bodies are indicative of apoptosis. (B) PANC-1 cells were incubated with Annexin V dye to verify apoptotic induction caused by NSC 51046. Cells were counterstained with Hoechst 33342 dye and propidium iodide (PI) to visualize nuclear morphology and cell death. Images shown are representative of two independent experiments at 48 hours following treatment with NSC 51046 and colchicine.

**Figure 4:**
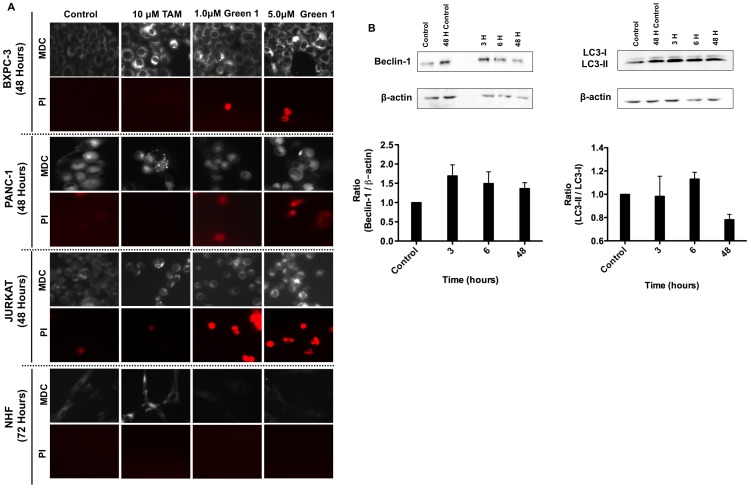
Green 1 Induces Selective Pro-Death Autophagy. (A) Pancreatic cancer cells, human leukemia cells (Jurkat) and NHFs were incubated with Monodansylcadaverine (MDC) to stain autophagic vacuoles and propidium iodide (PI) for cell death to determine if autophagic induction led to cell death. Bright, punctate staining is indicative of autophagy, as seen in our positive control (Tamoxifen treated cells). Images were taken at 400X magnification on a fluorescent microscope. (B) Western blot analyses of Beclin-1 expression and LC3 conversion were conducted on PANC-1 cells treated with 5.0 µM Green 1 at various time points. β-actin was measured as an internal control. Samples treated for 3 and 6 hours were compared to the early control, while the sample treated for 48 hours was compared to the 48-hour control. Results are expressed as mean ± SD from two independent experiments for both Western blots.

To investigate the role of allocolchicine derivatives on cell death induction in cancer cells, the nuclei of cells were stained with 10 µM Hoechst 33342 dye for 10 minutes following treatment with either Green 1 or NSC 51046 for either 48 or 96 hours. In the resulting images (Fig. 3A), it was observed that NSC 51046 treatment led to nuclei which exhibited apoptotic morphology, including cell blebbing, shrinkage and condensed nuclei for both pancreatic cancer cell types and the non-cancerous normal fetal fibroblasts (NFFs), corresponding to the viability data. However, cancer and non-cancerous cells treated with Green 1 did not show this apoptotic morphology, suggesting that Green 1 does not induce apoptosis in pancreatic cancer cells. The apoptotic induction by NSC 51046 was confirmed using an Annexin V binding assay. Annexin V binds to externalized phosphatidylserine, which is a marker of early apoptosis [28]. In the resulting images (Fig. 3B), it was found that treatment with both doses of NSC 51046 lead to the externalization of phosphatidylserine as shown by the green fluorescent staining. Furthermore, it was also observed that treatment with colchicine induced apoptotic cell death as it led to nuclear fragmentation, externalization of phosphatidylserine and cell death. The lack of apoptotic morphology and the reduction in the viability of pancreatic cancer cells treated with Green 1 led us to investigate other modes of cell death. Following treatment, cells were then incubated with monodansylcadaverine (MDC) to determine if there was autophagic induction. We observed the induction of autophagy at 48 hours in all cancer cells tested, comparable to autophagic induction by our positive control for pro-survival autophagy, Tamoxifen [10] (Fig. 4A). Furthermore, staining with propidium iodide (PI) revealed that, unlike Tamoxifen, Green 1 treated cancer cells were positive for propidium iodide and exhibited a pro-death form of autophagy (Fig. 4A). This activity of Green 1 was specific to cancer cells, as NHFs treated with Green 1 did not stain positive for the presence of autophagic vacuoles with MDC or necrotic cell death with PI, confirming the selectivity of Green 1 to cancer cells. In order to confirm the induction of autophagy caused by Green 1, Beclin-1 expression and LC3-I to LC3-II conversion were assessed by western blot analyses in PANC-1 cells treated with 5.0 µM Green 1 for various time intervals (Fig. 4B). β-actin was measured as an internal loading control. Densitometry was performed and samples treated for 3 and 6 hours were compared to the early control, while the sample treated for 48 hours was compared to the 48-hour control. The resulting graphs show that Green 1 treatment caused an increase in Beclin-1 levels at all time points following treatment. LC3-II levels were moderately increased at 6 hours following treatment with Green 1.

### Intracellular Targets of Green 1

**Figure 5:**
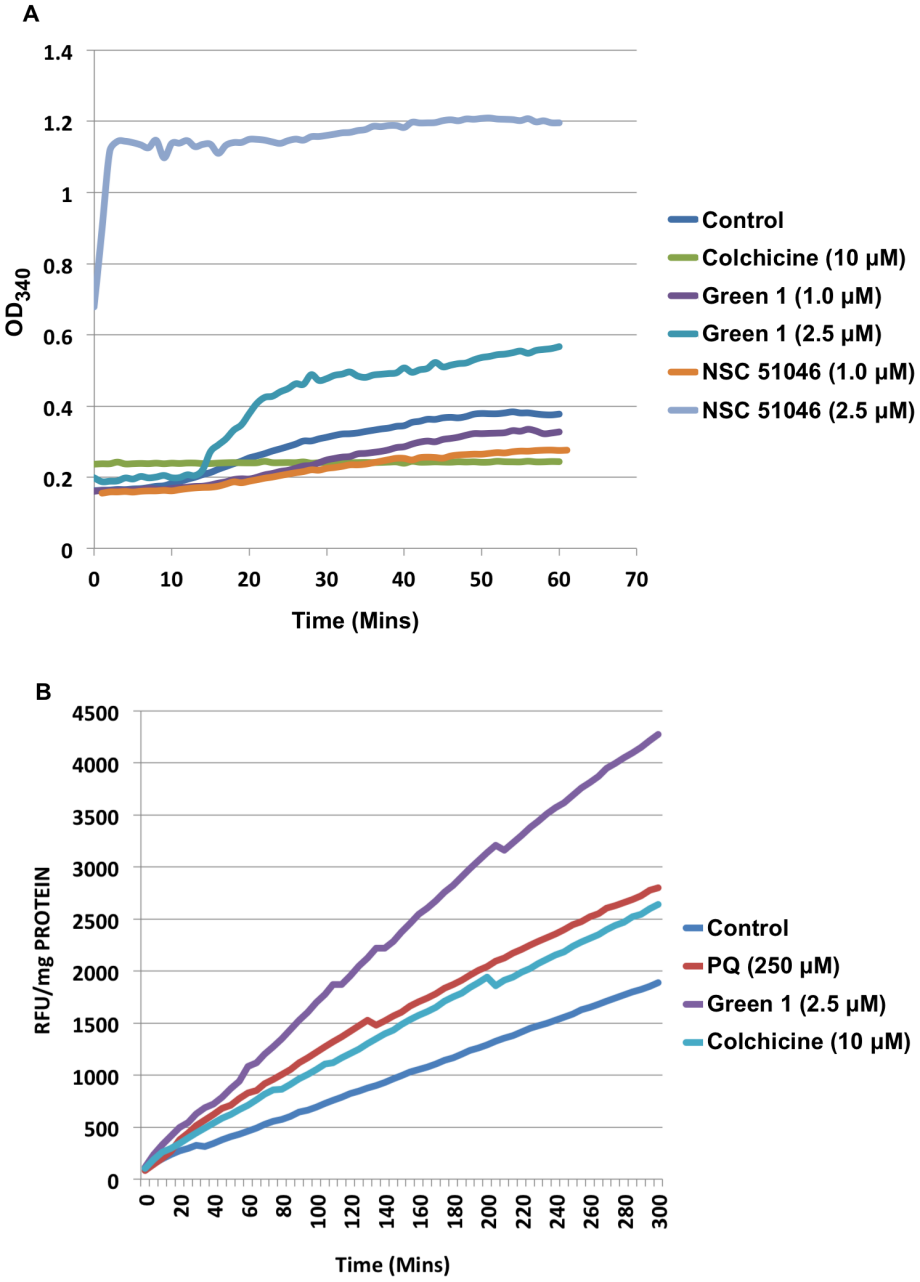
Intracellular Targets of Colchicine Derivatives. (A) Colchicine derivatives (Green 1 and NSC 51046) were incubated with porcine tubulin for an hour in a 96-well plate. The absorbance (OD _340 nm_) was obtained every minute during the one hour incubation. Colchicine was used for comparison to the derivatives. (B) Isolated mitochondria from PANC-1 cells were treated with Green 1 and ROS production was measured using Amplex Red substrate in the presence of horseradish peroxidase (HRP). Results were compared to control untreated mitochondria, colchicine treated mitochondria and positive control, paraquat (PQ). Fluorescence readings were taken in 5 min intervals for 4 hours at Ex. 560 nm and Em.590 nm and expressed as relative fluorescence units (RFU). Results are a representative of three independent experiments.

Colchicine and some of its derivatives are known to induce apoptosis in cancer cells by inhibiting tubulin polymerization and leading to DNA damage, which acts as a signal for the initiation of the process of apoptosis [29]. As we observe the differential effects of Green 1 and NSC 51046 in cell death induction, we wanted to further investigate the potential intracellular targets of Green 1, in comparison to NSC 51046 and colchicine. In particular, the effects of colchicine and its derivatives on tubulin polymerization were evaluated. Green 1, NSC 51046 or colchicine were incubated with tubulin purified from porcine brain. The OD_340_ was measured every minute for one hour and the resulting graph is shown in Figure 5A. Our results indicate that NSC 51046 induced slight tubulin inhibition at the lower dose, which corresponds to previous studies [31], [32]; however, we observed that a higher dose of NSC 51046 caused rapid tubulin polymerization, which was unexpected. As expected, colchicine inhibited tubulin polymerization. Treatment with Green 1 at higher doses led to a slight increase in tubulin polymerization, while the lower Green 1 dose remained relatively close to the control sample (Fig. 5A). This confirms that Green 1 and NSC 51046 have distinct biochemical mechanisms, as NSC 51046 targets tubulin polymerization to a much greater extent than Green 1. These results suggest that a simple change in the structures of these allocolchicine analogues has led to very different biological activities in normal and cancerous cells.

### Green 1 Causes Increased ROS Production in Isolated Mitochondria

Mitochondria are key players in the initiation and progression of cell death processes and thus, serve as potential targets for many chemotherapeutic agents [30]. To determine if the mitochondria is involved in Green 1-induced cell death, mitochondria were isolated from pancreatic cancer cells (PANC-1) and directly treated with colchicine, Green 1, or paraquat (PQ, positive control), for 5 hours or left untreated. The fluorescence was measured every 5 minutes throughout the experiment to quantify the levels of reactive oxygen species (ROS) production. Green 1 induced a substantial increase in ROS production, even greater than the positive control, PQ and colchicine (Fig. 5B). This further substantiates that the structural change in the allocolchicine derivatives has led to distinct mechanisms.

### Green 1 is Well - Tolerated in Mice

**Figure 6:**
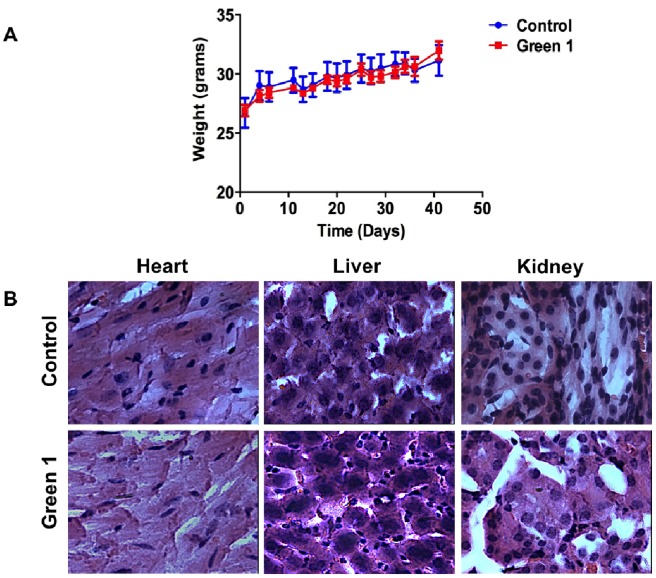
Green 1 is Well-Tolerated in Mice. Following acclimatization, CD-1 nu/nu mice were separated into two groups, one group was injected subcutaneously in the right and left hind flanks with Me_2_SO in PBS (10 µL in 200 µL PBS), while the second group received subcutaneous injections of Green 1 (10 mg/kg/day for a total volume of 10 µL Green 1/200 µL PBS) three times a week for a period of one month. (A) Mice weights were recorded twice a week for the duration of the study. (B) Following the study, mice were sacrificed and their organs were obtained for histopathological assessment. Hematoxylin and Eosin staining was carried out on the tissue samples.

Due to the intracellular targets of allocolchicines and some of its derivatives, like ZD 6126, there have been documented toxicities and significant side effects associated with their use. Specifically, cardio-toxicity in humans was exhibited during clinical trials of ZD 6126 [23]. To further characterize the selectivity of Green 1 and its potential toxicity, *in vivo* studies were carried out. To do this, immunocompromised CD-1 nu/nu mice were obtained from Charles River laboratories and following acclimatization, mice were divided into two groups, one group received vehicle injections (5% DMSO in PBS) and the other group was treated with Green 1 at a dose of 10 mg/kg/day, 3 times per week, for a total of 41 days and 15 treatments. Mice were weighed three times a week for the duration of the study. The weight changes between the control and the Green 1 treated group were comparable, with no loss in weight observed in any of the treatment groups (Fig. 6A). Following the treatment period, mice were sacrificed and their organs (liver, heart and kidneys) were obtained for pathological analysis by hemotoxylin and eosin (H&E) staining. The resulting images (Fig. 6B) show that there are no gross morphological differences between the groups. This confirmed that Green 1 shows no toxicity in *in vivo* studies and is well tolerated in animals.

## Discussion

In this report, we describe the potential anticancer activity of a novel allocolchicine derivative, (*S*)-3,8,9,10-tetramethoxyallocolchicine, which we refer to in this study as Green 1. Here we have shown that Green 1 modestly reduced the viability and induced a pro-death form of autophagy selectively in pancreatic cancer cells (Fig. 2A, 4A and 4B), with no apparent effect on normal non-cancerous fibroblasts (Fig. 2A and 4A). Furthermore, we observed an increase in pro-death autophagy following treatment with Green 1 in leukemia cells (Fig. 2B and 4A). As these rapidly dividing leukemia cells are growing for 96 hours, these cells quickly use up the nutrients available and produce high levels of metabolic waste, thus producing the 20% basal level of cell death. However treatment with Green 1 led to an additional 40% increase in cell death in the treated cells. We also report that Green 1 did not have a significant effect on tubulin polymerization or cause apoptosis (Fig. 3A and 5A), unlike colchicine and some of its other derivatives [23]. However, we observed mitochondrial targeting, as treatment of isolated mitochondria led to increased ROS production (Fig. 5B). Our *in vivo* studies revealed that Green 1 is well tolerated in mice at a dose of 10 mg/kg/day, as observed by the lack of change in the weights, both in the control and the Green 1 treated groups (Fig. 6A). Moreover, there was no distinct difference in the levels of protein in the urine of mice, collected from the control and the Green 1 treated group (data not shown). Furthermore, we found no gross morphological changes in the H & E stained tissues (hearts, livers and kidneys) obtained from mice, following the duration of the study (Fig. 6B). Interestingly, another colchicine derivative with a slightly different chemical structure, N-acetyl-O-methylcolchinol (NSC 51046) (Fig. 1), caused non-selective apoptosis and induced rapid tubulin polymerization at high doses. Collectively, the results suggest that a small change in the chemical structure of colchicine derivatives could lead to very distinct biochemical mechanisms and different activities. It should be noted that the results from the WST-1 assays, trypan blue viability assays and fluorescence microscopy were very similar; Green 1 induced autophagic cell death in approximately 20% of the pancreatic cancer cells, 60% of leukemia cells and had little to no effect on the normal human fibroblasts, while NSC 51046, on the other hand, caused apoptotic cell death in approximately 70% of both the pancreatic cancer cells and normal fibroblasts.

As mentioned above, colchicine and many of its derivatives (ZD 6126) target tubulin. In fact, many of the current chemotherapeutics available target tubulin or other DNA replicative machinery as a means of killing cancer cells [24]. It is well established that compounds that target DNA replicative machinery affect not only the fast dividing cancer cells, but also fast dividing non-cancerous cells in the body, leading to therapy associated side effects with common chemotherapy. NSC 51046 is known to inhibit tubulin polymerization at low doses [31], [32], which we also observed; surprisingly, it was observed the NSC 51046 induced rapid tubulin polymerization at a higher dose. NSC 51046 was also observed to be non-selective, further illustrating that tubulin is not an optimal target for cancer targeting drugs. This rapid tubulin polymerization may be the reason for the non-selective properties of NSC 51046. Green 1, on the other hand, caused slight tubulin polymerization at the higher dose but not to the same extent as NSC 51046. Furthermore, we found that treatment with Green 1 led to increased ROS production from isolated mitochondria (Fig. 5B) so as to induce pro-death autophagy selectively in cancer cells. This poses the question: Why are only the cancer cells undergoing necrosis and autophagy?

**Figure 7:**
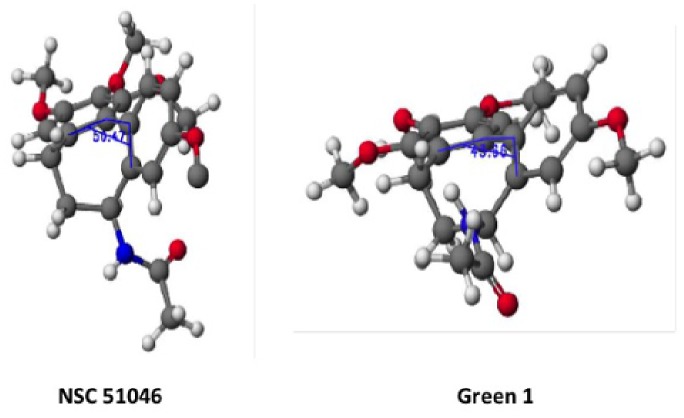
DFT Structures of NSC 51046 and Green 1.

Perhaps the answer lies in the differences between the mitochondria of normal cells and cancer cells. Since cancer cells proliferate rapidly and uncontrollably, many tumors are subjected to hypoxic conditions, leading to more vulnerable cancer cell mitochondria that do not produce enough ATP to satisfy the energy needs of the cells [33]. In part, this consequently leads to an increased reliance on glycolysis for ATP generation, known as the Warburg effect [34]. The increased ROS production caused by Green 1, points to the mitochondria as a possible target. Furthermore, we predict that the pro-death autophagy is the result of increased oxidative stress and damage caused by treatment with Green 1. This oxidative stress leads to the induction of autophagy, possibly as a mechanism to survive the stress of exposure to Green 1. However, the cells are unable to survive the continued exposure to the compound, ultimately succumbing to cell death due to the induced autophagy. Structurally, our working hypothesis centers around the effect of the rearranged methoxy groups on the biaryl dihedral angle. It is well established that the presence of the seven membered B ring is key for activity in the allocolchicines [32]. This imposes dihedral angles for the biaryls in the 49–53 ^o^ range [35], [36], which appear to be near optimal for tubulin binding. The lack of a methoxy function in Green 1 at one site ortho- to the biaryl axis reduces this dihedral angle; based on DFT calculations (CAChe® v. 6.1.12.33, B88-PW91 functional, dzvp basis set), this reduction is from 50.5 ^o^ in NSC 51046 to 44.0 ^o^ in Green 1 (Fig. 7). ^1^H NMR spectroscopy also strongly suggests a reduced barrier to atropisomerism in Green 1 [25].

These results open a new window of possibility. We have shown that a slight change in the chemical structure of a compound can lead to drastically different biological properties. This validates the synthesis of novel analogues of known compounds. Although Green 1 is not potent enough to act as a cancer therapy alone, we hope that our structure activity relationship study can provide insight towards developing new allocolchicine analogues with greater selective anticancer activity.
